# Renal Transplantation-Anaesthetic Experience of 350 Cases

**Published:** 2009-06

**Authors:** Anand Jain, Vaibhavi Baxi, D Dasgupta

**Affiliations:** 1,2 Clinical Associate, Department of Anaesthesia, Jaslok Hospital and Reserch Center, Mumbai; 3 Head of the Department, Department of Anaesthesia, Jaslok Hospital and Reserch Center, Mumbai

**Keywords:** Renal transplantation, End stage renal disease, General anaesthesia

## Abstract

**Summary:**

Transplantation provides a near normal life and excellent rehabilitation compared to dialysis and is the preferred method of treatment for end stage renal disease patients. We describe our experiences through a retrospective analysis of anaesthesia management of 350 cases of both living related and cadaveric renal transplantation conducted between Jan 2004 - April 2008 at Jaslok Hospital And Research Center. Areas of our interest include preoperative patient status, fluid management, hemodynamic stability, anaesthesia management, and perioperative complications. Recent advances in surgical techniques; anaesthesia management and immunosuppressive drugs have made renal transplantation sale and predictable. Preoperative patient optimization, intraoperative physiological stability and postoperative care of renal transplant patients have contributed to the success of renal transplant programme in our hospital.

## Introduction

Organ viability associated with renal transplantation is a product of the managing of the donor patient, the allograft, and the recipient patient. Short- and long-term outcome is influenced by perioperative fluid and drug treatment, and the function and viability of the transplanted kidney seems to be optimized if graft perlusion is maximized through mild hypervolemia. At the same time careful balancing of intraoperative fluids is necessary against cardiovascular problems frequently encountered in patients With uremia. Close intraoperative monitoring, optimization of intravascular fluid volume status to maximize kidney perfusion, and prompt correction of electrolyte disturbances (especially potassium) are key to short- and long-term success of renal transplants.^1^

We conducted a retrospective analysis of 350 cases of living and cadaveric renal transplants to identify the trends according to patient's age, sex, cause of chronic kidney disease (CKI)), anaesthesia management and the outcome of patients in our hospital.

## Methods

**Fig 1:**
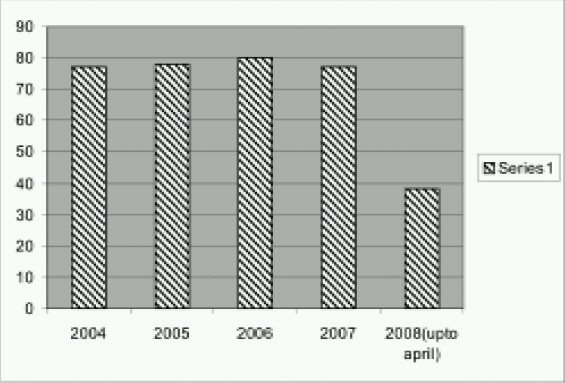
No. of renal transplantations per year at Jaslok hospital

In this retrospective study we reviewed medical records of 350 cases of living and cadaveric kidney transplants conducted from Jan 2004 - April 2008 at Jaslok Hospital And Research Center, Mumbai. (Fig 1) As per the hospital policy all drugs used and events that occur pen operatively were recorded manually and a copy of the preoperative assessment and anaesthesia notes written by the concerned consultant anaesthesiologist were preserved. We noted age, sex, type of transplant, cause of CKD, preoperative status, and history of dialysis. Preoperative preparations and investigations, details of anaesthesia management and monitoring and the outcome were also recorded and entered into an electronic database.

Live related donors (336 patients) belonged to ASA grade 1 and 2. All living donor nephrectomies; including a few laparoscopic nephrectomies were conducted under general anaesthesia with controlled ventilation. Our study though focussed on the management of the recepients of renal transplant.

## Results

350 patients who underwent renal transplantation at the study hospital from Jan '04 to April '08 were in the age bracket of 10 to 70yrs, with median age being 30 to 40yrs.

**Fig 2:**
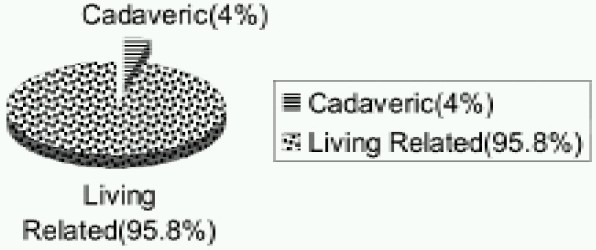
Type of renal transplantation

Of the total no.of patients 14 (4%) underwent cadaveric renal transplant while the rest (96%) underwent living, related renal transplant. (Fig 2).

**Fig 3:**
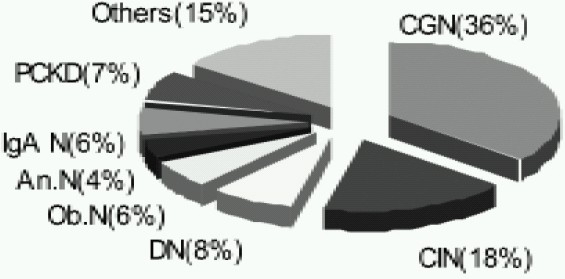
Causes of end stage renal disease

There was predominance of male patients with 268 (76%) males and 82 females (24%).24 (6.8%) patients were pediatric. The causes ofend stage renal disease (ESRD) were chronic glomerulonephritis (CGN)-36%, chronic interstitial nephritis (CIN) 18%, diabetic nephropathy (DN)-8%, polycystic kidney disease (PCKD)-7%, obstructive nephropathy (Ob.N)-6%, IGA nephropathy-6%, analgesic nephropathy (An.N)-4% and in 15% patients other causes were noted. (Fig 3).

### Preoperative status

Of the 350 patients, 337 (96.3%) patients had been on hemodialysis while 13 (3.7%) patients had been on continuous ambulatory peritoneal dialysis (CAPD). Anemia was a common finding in most of the patients. Average hemoglobin (Hb) was 7.6gm% (hematocrit-22.8). Hb was less than 7.6gm% in 18% of the patients. Iron supplements were given to 85.1% patients, while 6% received erythropoietin and 13.1% underwent preoperative blood transfusion. Serum potassium levels were in the range of 4 – 6.8 meq/1 with an average of 4.9meq/1(±0.87SD). Serum creatinine levels were in the range of 3.4 – 19.1mg/dl with an average of 9.4mg/d1 (±3.45SD). Electrocardiogram of some patients had left ventricular hypertrophy (93), tall T wave (18), ST-T abnormalities (14), left atrial enlargement (9) and right bundle branch block (6). Ejection fraction was <20% in 27 patients and <30% in 59. Pericardial effusion was present in 20 patients and 7 had left ventricular thrombus on preoperative 2 D echo. On chest X-ray 47 patients had bilateral pleural effusion. Human leukocyte antigen (HLA) match between donor and recipient tissue was done in all patients.

Three-drug regimen of immunosupressants including methylprednisolone was used to decrease the incidence of graft rejection. Their use is divided in three phases –first phase of induction therapy before and during first week post transplant and involves marked immune suppression. The second phase is the maintenance therapy involving, drug administration continuously for three to six months to prevent acute graft rejection. They also induce tolerance. The third phase involves long-term immunosupression maintained for the rest of the life. The following drugs were used for immunosupression -

Steroids, Cyclosporin, Tacrolimus, Sirolimus, Antilymphocyte globulin, Basiliximab, OKT 3 and Azathioprine. 94% recipients were on oral anti hypertensives drugs, which Were continued on the morning of the surgery.

### Anaesthesia management

General anaesthesia was the technique of choice in most (345) of the cases. Continuous epidural anaesthesia with intermittent intravenous sedation was used in 5 cases.

At the study institution all living related renal transplants were done electively while cadaveric renal transplants were done in emergency or semi-electively. Hemodialysis was performed in almost all recipients within 24 hours before surgery to reduce the risk of volume overload, hyperkalemia, and excessive bleeding. Pre-medication one hour before surgery consisted of ranitidine hydrochloride 150mg orally in addition to all other medications that the patients were receiving on a regular basis.

Peripheral intravenous access was secured in the hand opposite to the functioning fistula and induction of anaesthesia was done with propofo1(2mg.kg^−1^) in 303 (86%), thiopentone(5mg.kg^−1^) in 42 (12%) and with etomidate(0.2mg.kg^−1^) in 7 (2%) patients. Neuromuscular blockade was maintained with either atracurium [0.6mg.kg^−1^] (290), or rocuronium [0.6mg/kg] (51) orvecuronium [0.1 mg.kg^−1^] (9). All patients were intubated and ventilated. Anaesthesia was maintained with 40% N_2_O in oxygen supplemented with 1-2% isoflurane (276)/ 1-2% sevollurane (74) with fresh gas flow of 2 l/min. Analgesia was maintained with fentanyl 2-5mcg.kg^−1^ (l44) or pentazocine 0.5mg.kg^−1^ (153) or Morphine 0.l mg.kg^−1^ (53).

Intraoperative monitoring included heart rate, non-invasive blood pressure, oxygen saturation, end tidal CO_2_ and electrocardiogram in all patients. Central venous line was placed in the right or left internal jugular vein (depending upon the presence oldialysis catheter) in 53 patients for central venous pressure (CVP) monitoring and in 15 patients blood pressure was monitored invasively. Average duration of surgery was 5.5hrs(±1.30SD) and during this period intra venous fluid administered was normal saline-based crystalloid (NS, DNS) or colloid (gelofusine). 306 patients were transfused with only crystalloid while 44 patients received a combination of both crystalloid and colloid. 46 patients required intraoperative blood transfusion (packed cells). In the group of patients who required blood transfusion; the preoperative Hb was <7.6gm%. Intraoperatively 298 patients (85%) received injection furosemide average 40mg±23.09SD (1 to 1.5mg/kg).

Total ischaemia time noted was on an average 51.6 min(±12.29SD) with min-21 min and max of 94 min. For every one-minute of warm ischaemia time ten minutes of cold ischaemiatime was permitted. Kidney Would be placed in ice slush with a continuous perfusion of cold saline.

**Fig 4:**
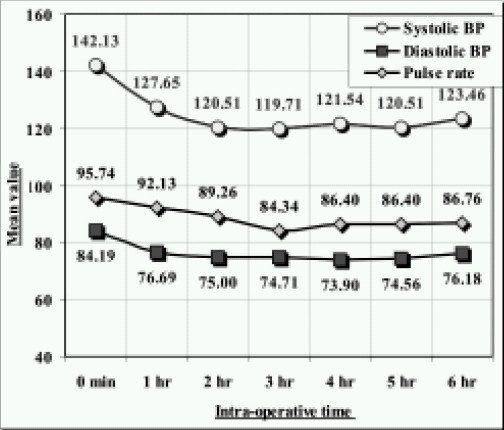
Systolic, diastolic B.P. and Pulse rate at various intraoperative time intervals

Hemodynamic parameters were recorded on hourly basis from the intraoperative charting. (Fig 4) Intraoperatively dopamine infusion at 2-5 microgram/kg was used in 40 patients. 12 patients had intraoperative artythmias in the form of premature ventricular contractions.

At the end of the surgery, neuromuscular blockade was reversed with neostigmine 0.05mg/kg, and glycopyrrolate 8mcg/kg intravenously. Most of the patients were extubated immediately postoperatively though recovery was delayed in 27 patients and 7 patients needed postoperative ventilatory support.

### Postoperative care

Patients were transferred to post kidney transplant care unit. Rescue analgesia was provided with tramadol (50-100mg) in 214 patients and both tramadol and pentazocine (25-30mg) in 130 patients. Epidural analgesia was used in 7 patients. Dialysis support was needed in 21 (6%) patients in the postoperative period. Acute tubular necrosis (ATN) developed in 54 patients, 12 developed pneumonia and 29 developed pulmonary edema. Acute graft rejection was seen in 13 patients, which responded to thymoglobulins and immunosupressants. Re-exploration was needed in 13 patients either for hematom a in the allograft or for the thrombus in vessels of the graft. Heparin infusion was started in 8 patients for 3 - 5days. 6 patients underwent nephrectomy. Postoperative mortality (over six months postoperatively) of 12 patients was seen in these 350 patients. (Table 1) The rest of the patients received life-long triple drug immunosupression.

**Table 1:** Postoperative complications

S.No.	Complication	No.of patients
1.	Acute tubular necrosis	54
2.	Acute respiratory distress syndrome	12
3.	Pulmonary edema	29
4.	Acute graft rejection	13
5.	Re-exploration	13
6.	Nephrectomy	6
7.	Mortality	12

## Discussion

Kidney transplantation is the treatment ofchoice for patients with end-stage renal disease.^2^ It is more cost effective than maintenance dialysis and usually provides the patient with better quality of life.^3^ Any surgical procedure, in patients with CKD has a significant increase in the perioperative morbidity and mortality. Preoperative work-up and intraoperative management of patients with end-stage organ disease are certainly among the most difficult and challenging areas in anaesthesia. Understanding the myriad alterations in physiology and function, both locally and systemically, is critical to providing safe and successful perioperative management. Co-morbid disease is common and frequently severe in these patients.

Organ transplantation at Jaslok Hospital currently consists of renal and hepatic transplants. Of these, renal cadaveric and living donor transplants are by far the most common. This audit shows that the renal transplantation programme at this institute has been successful. Factors responsible for good outcome are proper planning and team efforts by all concerned in the transplant team in addition to good preoperative preparation of the patient. Preoperative control of the systemic effects of CKD in recipients, well controlled intraoperative hemodynamics, and good postoperative medical care resulted in success of the transplant programme.

In our experience pediatric renal transplant have been associated with better outcome. This corroborates with the findings from studies on pediatric transplants in the UK and North America^4^ who have reported better long-term graft survival rates in children as compared to adults.^5^

Fluid management remains a controversial subject in perioperative medicine and organ transplantation. Recent advances in the understanding of pharmacokinetic and pharmacodynam ic profiles of fluids, as well as transplantation physiology and pathophysiology, can guide us in new approaches to common problems. Fluid therapy in transplant medicine is usually best practiced using goal-directed approaches and balanced electrolyte formulations when possible. Crystalloid solutions are usually preferred to correct fluid and electrolyte imbalance. In our study, majority of patients (87%) were transfused with normal saline-based crystalloids; as most anaesthesiologists would avoid potassium containing fluids during renal transplantation, with the belief that it may worsen the hyperkalemia in the event of impaired graft function. However in a recent randomized, double blind study comparing Ringer's solution and 0.9% normal saline during renal transplant the authors have shown those who received Ringer's solution had less hyperkalemia and acidosis. The saline infusion leads to acidosis possibly by dilution of bicarbonate by large volumes of buffer free fluid or the resultant hyperchloremia decreases the strong ion difference with the development of acidosis. The hyperchloremic metabolic acidosis leads to hyperkalemia by shilt of potassium into extracellular space. However in this study the average volume of crystalloid infused during the surgery was about 6 litres.^6^

Both crystalloids and colloids have been used for volume replacement. Over the last few decades there has been a shift in practice from using natural colloid to synthetic colloids. There has been some concern regarding the use of hydroxyethyl starch (HES) as osmotic, nephrosis-like lesions were demonstrated in transplanted kidneys retrieved from deceased donor who were transfused with HES200/0.62^7^. In our study 44 (12%) patients received a combination of crystalloids and colloids with the colloid being mainly gelatin based. Some fluids may exert drub effects that could alter organ preservation and reperfusion, while the low molecular weight HES appears to be less toxic in renal transplantation than first suspected, especially when clinicians consider free water requirements in these settings.^8^

Standard ASA monitors Were used in all the patients, however in patients with more advanced stages of co-morbid conditions, more extensive monitoring such as CVP monitoring (53patients) or continuous arterial pressure monitoring (15patients) were used. Inotropic support was used in 40 patients. No major rise in blood pressure was seen in these patients. In a large series of renal transplantation by Heino H and Orko R hypotension (49%) was a more common finding than hypertension (26.8%).^9^

A retrospective study of postoperative respiratory morbidity in 247 patients requiring renal transplantation showed that 7 patients required postoperative controlled ventilation. Long acting non-depolarising relaxants were used in only 65 patients, but all 7 cases of respiratory failure occurred in this group, which suggests that the use of these drugs in anephric patients is potentially hazardous so far as postoperative respiratory insufficiency is concerned.^10^ In an other study by Avner Sidi and Richard Kaplan, prolonged neuromuscular blockade has been reported in 8 out of 65 patients of renal transplant who had received either Vecuronium(4 Out of 29) or atracurium(4 out of 36).^11^ In our study too neuromuscular blockade was maintained with either atracurium (290) or rocuronium (51) or vecuronium (9). Persistent neuromuscular blockade with delayed recovery was seen in 27 patients and 7 patients needed postoperative ventilatory support. In our study only 5 cases were done using continuous epidural anaesthesia with intermittent intravenous sedation. No major complication was reported in either of these cases. There have been reports of renal transplants done under continuous epidural anesthesia with intermittent sedation with intravenous agents by Lauretti, Gabriela Rocha. They also reported frequent respiratory complications and intraoperative rupture of the renal anastomosis due to cough, hiccups and agitation. As an alternative technique, the laryngeal mask airway (LMA) was used to maintain clear upper airways during continuous epidural anesthesia.[12]

Transplant anaesthesia is a specialized field, which requires a good understanding of the abnormalities in patients with renal failure, familiarity with transplant medicine and expertise in management of these patients. With improvement in anaesthetic and surgical techniques as well as immunosuppressive drugs, many patients are being accepted for transplantation who would have been considered unsuitable earlier. Proper patient selection, preoperative patient preparation and intraoperative physiological stability with close association between nephrologist, urosurgeons and anaesthesiologists have found a valuable place in the management of our renal transplant patients and has given us good results. Further large scales studies are desired.

## Preparation of the Manuscript

The text of original articles should be divided into sections with the headings: Summary, Key-words, Introduction, Methods, Results, Discussion, References, Tables and Figure legends. For a brief report include Summary, Key-words, Introduction, Case report, Discussion, Reference, Tables and Legends in that order. Do not use subheadings in these sections. Use double spacing throughout. Number pages consecutively, beginning with the title page.

**Table:** 

	*Abstract Word Length*	*Maximum Text Word Length*	*Maximum No. of Figures/Tables*	*Maximum No. of References*
***Review Article***	250	4000	8	90
***Special Article***	250	3500	5	50
***Clinical Investigation***	250	3000	5	30
***Case Report***	100	1000	3	10
***Letter to Editor***	N/A	500	1	5

**Pramila Bajaj**

Editor, IJA

Email: bajajpramila@hotmail.com
